# Engaging vs. Non-Engaging Abutments: An In Vitro Study Evaluating Changes in Microgap and Screw Morphology

**DOI:** 10.3390/dj12080265

**Published:** 2024-08-20

**Authors:** Fawaz M. Alzoubi, Mohammad Y. Sabti, Esra Alsarraf, Faris A. Alshahrani, Steven J. Sadowsky

**Affiliations:** 1 Department of General Dental Practice, College of Dentistry, Kuwait University, P.O. Box 24923, Safat 13110, Kuwait; 2 Department of Restorative Sciences, College of Dentistry, Kuwait University, P.O. Box 24923, Safat 13110, Kuwait; 3 Department of Substitutive Dental Sciences, College of Dentistry, Imam Abdulrahman Bin Faisal University, P.O. Box 1982, Dammam 31441, Saudi Arabia; 4 Preventive and Restorative Department, Arthur A. Dugoni School of Dentistry, University of the Pacific, San Francisco, CA 94103, USA

**Keywords:** abutments design, biomechanics, microgap, prosthetics, screw morphology, cyclic loading, abutments, prosthetic design, SEM, morphology

## Abstract

Background: The purpose of this study was to compare the microgap size between engaging (E) and non-engaging (NE) abutments and screw morphology changes between E and NE abutments using scanning electron microscopy (SEM) before and after cyclic loading (CL). Methods: Thirty-six implants were arranged into four groups as follows: Group 1, single units with E abutments; Group 2, single units with NE abutments; Group 3, three-unit fixed partial dentures with a hemi-engaging design; and Group 4, three-unit FPDs with two NE abutments. The microgap was evaluated using a stereomicroscope. SEM was used to qualitatively evaluate screw morphology. The specimens were subjected to axial loading first and then lateral loading (30°) using the settings; one million cycles (1.0 × 10^6^ cycles) for each loading axis. Results: There were no significant differences detected in the microgap sizes between the E and NE abutment groups. In addition, there were no significant changes in the microgap sizes after CL in the E or NE abutment specimens. More damage to the screws was noticed after CL compared to before, with no difference in the patterns of damage detected between the E and NE abutments. Conclusions: No significant difference in microgap size was detected between the E and NE abutments. Furthermore, there was no significant difference in microgap size between the different prosthetic designs. From the SEM qualitative evaluation, there were similar screw morphology changes after CL between the E and NE abutments.

## 1. Introduction

The advent of dental implants to restore patients’ edentulous sites has been documented, and high survival rates have been shown [1]. Nonetheless, mechanical and biological complications have frequently been reported and are partly attributable to the assembly of the components [2,3,4]. The implant platform is designed to accommodate a prosthetic component, known as the abutment, which is secured into the implant by a screw. Hence, a connection is formed between that abutment and the platform of the implant. This connection is referred to as the implant–abutment interface (IAI). With the exclusion of one-piece implant systems, an unavoidable space is created within the IAI. This space is commonly referred to as the “microgap”. The size of this microgap reflects the marginal fit, which is an important factor in the stability of the screw joint and implant assembly [5]. Several factors influence the size of the microgap, including the system used [6,7], the design of the contact between the implant and abutment [8], casting procedures [9], the degree of screw torque [10], and the degree of tolerance between components [11,12].

Two categories of complications that are due to this microgap have been identified: mechanical and biological. The mechanical issues include screw loosening [11], abutment rotation and breakage [12,13], preload reduction [11,12,13,14,15], and possible implant failure [12,16,17,18,19]. The biological issues include peri-implant mucositis [20], peri-implantitis [21,22], and halitosis [6,23].

The marginal fit within the IAI and mechanical complications are interrelated. Higher stress in the crestal bone and connecting components may be the result of a compromised IAI [24,25]. This stress might be the result of a mismatch between the components, leading to a compromised screw joint [12,26]. On the other hand, microgap size can increase as a result of screw loosening and a compromised screw joint [20,27,28]. Therefore, high precision between components minimizes those forces, thereby avoiding mechanical complications [29,30]. These mechanical complications can be a result of micromotion within this complex during function [31,32]. The effect of micromotion can be reflected in screw performance as well as in changes in screw morphology [33,34]. The greater the microgap, the greater the micromotion and the greater the impact on screw morphology.

The microgap in the IAI can also act as a niche for oral microorganisms. As the microgap size increases, microbial penetration and colonization increase due to leakage, leading to inflammation around the peri-implant tissue [6,7,20,27,28,29,35,36], which results in the biological consequences mentioned previously. Bacteria-infiltrated connective tissue has been reported after an inflammatory response around the IAI, even in plaque-controlled patients with healthy tissue surfaces [37,38]. Furthermore, micromotion may increase microleakage around the IAI as a result of the “micropumping” effect [38,39,40].

Most studies on microgap size evaluation and the impact of micromotion were conducted using external hex implant systems or compared external hex systems with internal hex systems [5,7,13,15,39,41,42,43,44]. Compared with external hex implant systems, internal hex implant systems provide a stable and self-locking IAI [12,44]. In internal hex implant systems, a mating surface between the external aspect of the abutment and the internal aspect of the implant is expected to create a stable connection that might minimize microgap size and micromotion [12,45]. Because of this connection, it is suggested that internal implant systems have reduced vertical discrepancies in the IAI, improved screw stability due to improved load distribution within the implant, and improved resistance to micromotion due to an improved engagement [12,17]. Nevertheless, controversy persists regarding whether internal implant systems provide properties that are superior to those of external hex systems by reducing the microgap size [5,46]. Furthermore, abutment designs within the internal implant configuration might have a significant role in microgap size. Abutments within the internal implant configuration can be classified as engaging (E) or non-engaging (NE) depending on the presence of an anti-rotational component projecting from the apical portion of the abutment [47]. E abutments contain insertion orientation features or grooves to prevent rotational movement, whereas the NE abutment does not. It has been theorized that this E component aids in creating a cold weld between the abutment and the internal aspect of the implant and that it may play a role in decreasing stress around the screw [48,49,50,51,52,53,54,55]. However, mean strain values were not shown to be significantly different between the E and NE abutments [56]. Whether this phenomenon plays a role in microgap size and micromotion is still not clear.

Although it is reported that most conical connections significantly reduce leakage into and out of the internal aspects of the implant system [57,58] and provide improved resistance to micromotion [12,17], limited data are available comparing abutment designs in internal hex systems. The significance of the IAI has been reported in several studies [11,12,13,14,21,22,43,59]; however, to the best of the authors’ knowledge, no study has compared the influence of the anti-rotational components in internal hex systems on microgap size and the impact of micromotion by evaluating changes in screw morphology. The aims of this study are (1) to compare the microgap size between the E and NE abutments in internal hex implants, before and after cyclic loading (CL), and (2) to compare the screw morphology changes between the E and NE abutments using scanning electron microscopy (SEM) after CL. The null hypothesis is that there are no significant differences in microgap size and screw morphology between engaging and non-engaging abutments after CL.

## 2. Materials and Methods

### 2.1. Specimen Preparation

A total of 36 internal hex implants (Nobel Biocare Replace Conical Connection; Nobel Biocare AG, Kloten, Switzerland) measuring 4.3 × 10 mm were used. All implants were mounted on auto-polymerizing polymethyl methacrylate resin blocks embedded in the centers of cylindrical metal tubes measuring 10 × 31 mm, with 1 mm of the implant crest exposed. For the specimens with multiple implants, an auto-polymerizing polymethyl methacrylate resin block was used to splint the metal tubes together. After embedding the implants and the complete polymerization of the resin, the process of fabricating the prostheses was initiated.

The specimens were divided into 4 groups: Group 1: single units with engaging abutments (*n* = 6); Group 2: single units with non-engaging abutments (*n* = 6); Group 3: 3-unit fixed partial dentures (FPDs) with a hemi-engaging design [48] (1 E and 1 NE abutments) (*n* = 12); and Group 4: 3-unit FPDs with 2 NE abutments (*n* = 12). All the units used were screw-retained full metal crowns. For the FPD groups, a pontic was designed with similar abutment dimensions between the 2 mounted implants. All the units were designed in a similar manner with similar dimensions (BL: 7 mm, MD: 8 mm, inter-occlusal: 8 mm). A silicone key was fabricated for the single-unit and FPD designs to ensure replication of the dimensions in all the specimens. A high-noble alloy (Wilbond 52SF; Wilkinson Dental Alloys, East Hampton, CT, USA) was used to cast all the specimens using custom abutments (GoldAdapt; Nobel Biocare AG, Kloten, Switzerland). All the waxing and casting was completed by the same experienced technician for consistency. Lab screws were used during the process of specimen fabrication.

The implant assembly was attached to a solid board and held in place by a bench vice to prevent rotation of the specimen during torquing. The crowns were inserted into the mounted implants. A digital screw torque meter (MGT50; Mark-10 corporation, Copiague, NY, USA) was used to measure the screw torque values with decimal precision throughout the study. The fit of the 3-unit FPD groups was evaluated visually (using a microscope) and manually before torquing the screws. The abutment screws (conical connection clinical screw, Nobel Biocare, Kloten, Switzerland) were torqued to 35 Ncm, as per the manufacturer’s recommendation. To minimize the settling effect, the new abutment screws were torqued twice, 10 min apart [60].

**Figure 1:**
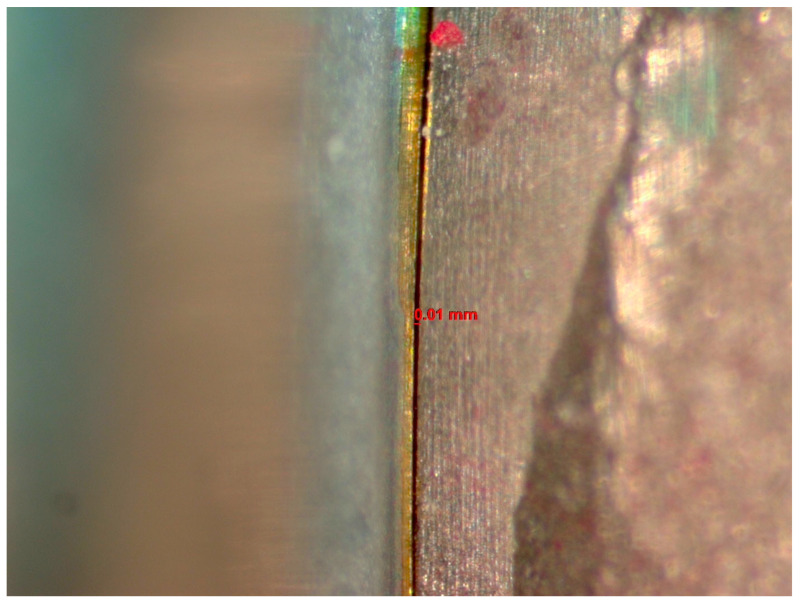
Image from the Discovery V12 stereomicroscope used for microgap evaluation. The line refers to the space measured.

Implant–Abutment Interface (Microgap) Evaluation:

The microgap was evaluated before and after cyclic loading using a stereomicroscope (Discovery V12, Zeiss, Jena, Germany) with 60× magnification and AxioVision software (SE 64-bit release 4.8.3, 09-2011). Three random sites were selected within the IAI to measure the sizes of the microgaps using a scale (Figure 1). The average size among the 3 sites was reported. Two investigators evaluated the IAI, and a measurement was established when both investigators reached an agreement regarding each reading.

### 2.2. Scanning Electron Microscopy (SEM)

**Figure 2:**
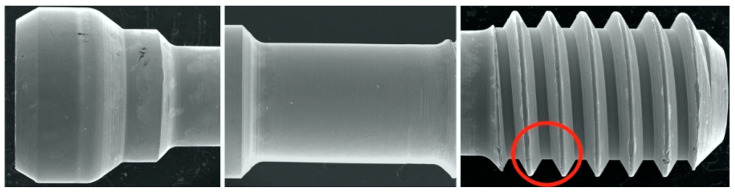
SEM images of sites evaluated. Red circle indicates thread site for qualitative evaluation performed and reported in Table 3.

SEM was used to qualitatively evaluate screw morphology before and after CL. Two specimens from each group were randomly selected for a baseline evaluation of the screw’s original state before torquing. A total of 12 screws were evaluated using a JSM-IT200 SEM machine (InTouchScope, JEOL, Tokyo, Japan) at 20.0 kV, as per the manufacturer’s instructions. Carbon tape was used to fix the screws on mounting plates. Three sites on the screws were selected for evaluation: the head, stem, and thread; the sites were evaluated at the following magnifications: 30×, 200×, and 500× (Figure 2). The surface features and threads of each screw were assessed subjectively by one blinded expert examiner for each of the following: homogenous vs. non-homogenous surface, smooth vs. striated, porous vs. non-porous, presence of surface debris, and any additional features that were notable, such as surface cracks or chips. Terms such as adhesive wear, galling, and plastic deformation were used to describe the different observations [61,62,63]. Adhesive wear was described as “the removal or displacement of material from a surface by the welding together and subsequent shearing of minute areas of two surfaces that slide across each other under pressure” [61,62,63], while galling referred to “a condition whereby excessive friction between two mating surfaces results in localized welding with a further roughening of the rubbing surfaces of one or both of the two mating parts” [61,62,63]. Additionally, the term plastic deformation was used to describe the alterations and changes in the original geometrical appearance of the screw surface or threads [62]. In addition to the previous descriptive terms, the term delamination was also used in this study to describe the appearance of the separation of the surface layer of the screw from the underlying surface. The term homogenous was used in the study by Guzaitis et al. to describe the screw surfaces [61]. In this study, this term was specifically used to describe any surface that showed a uniform appearance in the surface regardless of the exact nature of the surface. If the surface had uniform striation throughout the SEM image, it was described as homogenous. In contrast, if the surface displayed a non-uniform appearance, it was described as non-homogenous. Moreover, if the surface displayed obvious corrugations, it was considered striated, and if the surface lacked distinct striations, it was deemed smooth.

### 2.3. Cyclic Loading

All the groups underwent CL using the Electrodynamic Universal Testing Machine (ElectroPlus E3000, Instron, Norwood, MA, USA) with version 1.3 software. The specimens underwent axial loading first; then, after complete data collection from the axial CL, all the specimens’ components were checked visually and under the microscope to ensure that no significant damage was observed (excluding the abutment screws). Thirty-six new abutment screws were then used, and the specimens underwent lateral loading (30°) using the same CL settings used for the axial loading.

All the specimens were mounted firmly using a custom-made holding device. For groups 1 and 2, the load was aimed towards the screw access hole, i.e., the mid-occlusal area. For groups 3 and 4, the load was aimed towards the center of the mid-occlusal pontic area, including the connectors of the adjacent abutments. The load was applied to the units one million times (1.0 × 10^6^ cycles) for each loading axis (axial and lateral). Sine wave waveform CL with a force of 100 N was applied at a loading rate of 10 Hz. After each 180,000 cycles, the machine was stopped, and the specimens were carefully inspected for any movements or damage. No specimens showed any signs of movements or damage. CL was performed dry in a laboratory at 23 ± 1 °C and 50 ± 5% humidity. The effect of CL was assessed by evaluating any changes in the microgap size and screw surface morphology.

### 2.4. Statistical Analysis

A statistical analysis was performed using SPSS version 27 (IBM Corp. Released 2020. IBM SPSS Statistics for Windows, Version 27.0., Armonk, NY, USA, IBM Corp.). Descriptive information for the microgap sizes above 10 µm was presented as mean ± standard deviation values. After discovering minimal variation in the data, the microgap size was dichotomized as ≤10 µm versus >10 µm, and McNemar’s tests of symmetry were conducted to determine the significance of the microgap changes before and after CL. Chi-square tests were used to determine the significance of the differences in the dichotomized microgap sizes between the engaging and non-engaging abutments. A *p*-value of <0.05 was considered statistically significant. The methodology and statistics of this study were reviewed by an independent statistician.

## 3. Results

A total of 36 implants were used (12 single units and 24 FPDs), comprising 12 engaging abutments and 24 non-engaging abutments. No microgaps larger than 10 µm or smaller than 10 µm were observed in the specimens that underwent lateral CL. In addition, 50% or fewer abutments were observed with microgaps larger than 10 µm within the specimens that underwent axial CL. In order to maximize the possibility of finding any significant variation in microgap size based on CL or engagement, the data analysis focused exclusively on the abutments that underwent axial CL. Because the data did not have sufficient variation to analyze them as continuous, the microgap size was dichotomized (≤10 µm versus >10 µm) prior to comparative analysis. Table 1 presents descriptive statistics for the variation within the specimens that underwent axial CL and that were subsequently categorized as having microgap sizes over 10 µm.

Looking exclusively at the specimens that underwent axial CL, there were no significant differences detected in microgap size (≤10 µm versus >10 µm) between the engaging and non-engaging abutment groups, either before or after CL (Table 2). In addition, there were no significant changes in microgap size (≤10 µm versus >10 µm) in the E or NE abutment specimens before and after axial CL (Table 3).

**Figure 3:**
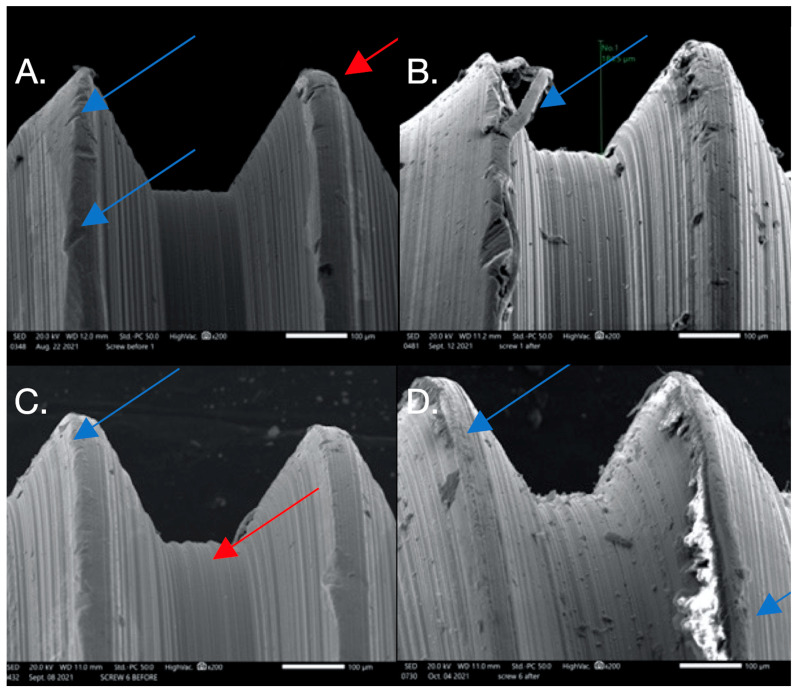
SEM images used to evaluate screw morphology. (**A**) Screw #1 before, showing a non-homogenous, striated, porous appearance as well as plastic deformation (surface damage) that could be the result of uncareful handling or a machining error in the component since this was before loading and placement. The screw on the thread flank is thinning compared to the other thread, creating a knife-edge appearance (blue arrow) that is also seen. Also noted is the presence of surface cracks (red arrow). (**B**) Screw #1 after: extensive surface damage and the separation of surface material of the thread, possibly caused by an adhesive wear mechanism or galling (blue arrow), which is described as “the removal or displacement of material from a surface by the welding together and subsequent shearing of minute areas of two surfaces that slide across each other under pressure”. (**C**) Screw #6 before: surface irregularities are present, probably due to mishandling or machining (blue arrow), and a notably striated surface is visible (red arrow). (**D**) Screw #6 after: surface chips and delamination and adhesive wear mechanism (galling).

**Figure 4:**
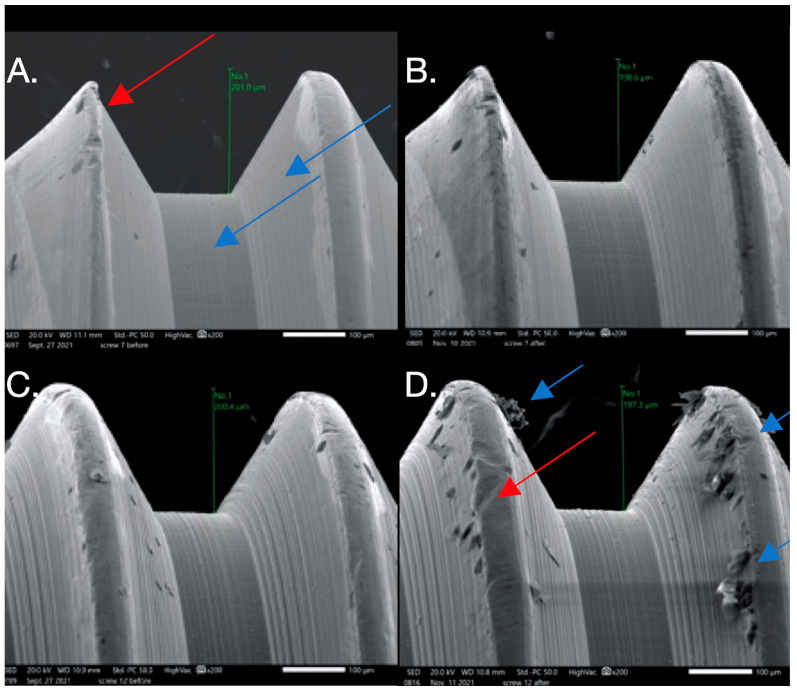
SEM images used to evaluate screw morphology. (**A**) Screw #7 before: note the smooth, non-striated surface vs. the previously striated surface on screws #1,6; it is non-porous. The thread appears to have plastic deformation where the thread is V-shaped (red arrow) compared to the adjacent thread, probably from the machining of the components. (**B**) Screw #7 after: no major changes are noted after loading. (**C**) Screw #12 before: it appears homogenous with a relatively smooth surface that has minimal striations, is non-porous, and has little surface debris that could be the result of machining of the components. (**D**) Screw #12 after: it appears non-homogenous (red arrow) with a considerable amount of surface debris and surface damage in the form of galling or adhesive wear (blue arrow). Changes in thread height were noted but were minimal.

**Figure 5:**
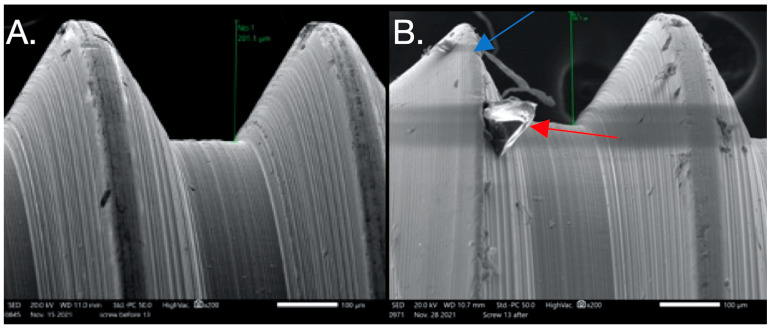
SEM images used to evaluate screw morphology. (**A**) Screw #13 before: the surface appears homogenous, with a striated appearance. (**B**) Screw #13 after: surface debris with adhesive wear (galling) and plastic deformation causing the thread to have a knife-edge appearance (blue arrow) and changes in thread geometry; a reduced thread height measurement was also seen. One area shows the gross separation of the surface material (red arrow). The surface of the screw appears to be smooth compared to its striated appearance before loading.

The images from the Discovery V12 stereomicroscope used for microgap evaluation are presented in Figure 1. SEM images obtained at 200× were analyzed in this study. The findings are summarized in Table 4 and Figure 2, Figure 3, Figure 4 and Figure 5. In general, more damage to the screws was noticed after CL compared to before CL, with no difference in the patterns of damage detected between the E and NE abutments. Nonetheless, an SEM analysis of some of the pristine screws before CL demonstrated signs of damage, such as non-homogeneity of surfaces, striations, porosity, and the presence of debris.

**Table 4:** SEM qualitative evaluation.

Group	Screw	Loading Condition	Homogenous	Non-Homogenous	Smooth	Striated	Porous	Non-Porous	Surface Debris	Additional Features
**1**	1 E *	Before		X		X	X			Plastic deformation+
		After		X		X		X	X	Plastic deformation + cracks + galling
	6 E *	Before		X		X		X		Surface irregularities
		After		X		X		X	X	Surface chips and delamination + galling
**2**	7 NE †	Before	X		X			X		
		After	X		X			X		
	12 NE †	Before	X		X			X		
		After		X	X			X	X	Surface chips + galling
**3**	13 E *	Before	X			X		X		
		After		X	X			X	X	Chips + galling + plastic deformation with thinning of the thread (knife edge)
	14 NE †	Before	X		X			X		
		After	X		X			X	X	
	23 E *	Before	X		X			X		
		After	X		X			X		
	24 NE †	Before	X			X	X		X	Surface irregularities
		After	X			X	X		X	
**4**	25 NE †	Before		X		X		X	X	
		After		X		X		X	X	
	26 NE †	Before		X		X	X		X	Surface irregularities
		After		X		X	X		X	Surface chips and irregularities
	35 NE †	Before		X		X		X		
		After		X		X		X	X	Surface chips + galling
	36 NE †	Before		X	X			X	X	Surface chips + irregularities
		After		X		X		X	X	Plastic deformation + chips + galling + cracks

* Engaging. † Non-engaging.

## 4. Discussion

In the present study, the authors investigated the influence of anti-rotational components in abutments within an internal hex implant configuration on microgap size by comparing the outcomes of the E and NE abutments before and after CL. The results of the present study suggest that there was no significant difference in microgap size between the E and NE abutments before and after CL. There were also similar screw surface morphology changes in the two abutment designs. Hence, the null hypotheses were accepted. Although there was a decrease in the microgap size after CL, this decrease in size was not statistically significant. The results also suggest that the difference in microgap size between the E and NE abutments after CL was not significant. Furthermore, the design of the prostheses (NE vs. hemi-engaging) did not impact microgap size before or after CL. It is worth mentioning that even though NE abutments are not used for single-unit prostheses, the investigators intended to use this design as a control to compare the results with their E counterparts and to provide a direct comparison. Furthermore, in this study, a hemi-engaging design was used to replicate what had been implemented in relatively recent practice [48]. It has been suggested that the use of a hemi-engaging design in a three-unit fixed, implant-supported prosthesis may improve the implant–abutment joint stability and minimize screw loosening in comparison with a more conventional NE design for both abutments [48,49,50,51,52,53,54,55].

A stable unaltered microgap within an IAI is critical to minimize both the biological and the mechanical complications mentioned previously. The impact on microgap size of a cold weld between the abutment and the implant in the E abutments needs to be revised. The present study suggests that the role of this cold weld is not significant in influencing the microgap size or improving the seal within the IAI and the micromotion, as reflected by the qualitative SEM evaluation of the screw surface morphology.

Most studies evaluating the microgap and IAI adaptation focused on external hex implants or on comparing external and internal implant systems [5,7,13,15,38,39,43]. The reported microgap sizes mainly ranged between 1 and 50 µm in the literature [29,64]. For the Branemark external implant system, Binon et al. [12] reported a 49 µm microgap. Ranges of 24.3 to 79.3 µm and 36 to 86 µm were reported by Dellow et al. [41] and Byrne et al. [13], respectively. Relatively, these are greater than the microgap reported in the present study. However, Tsunge et al. [5] reported mean vertical and horizontal discrepancies ranging from 22.6 to 62.2 µm and −27.1 to 16.0 µm, respectively, and microgap sizes of 2.3 to 5.6 μm, which are lower than the microgap sizes reported in the present study. Nonetheless, a clinically acceptable microgap was reported to be between 30 µm and 200 µm [65]. Inflammatory cells are expected to infiltrate microgaps exceeding 0.5 mm in size, leading to microleakage and bacterial colonization [20]. Tsunge et al. [5] also suggest that morphological features within the IAI had a direct effect on microgap size. In the present study, the GoldAdapt abutments had a sharp bevel margin, which is a variable that might have contributed to the relatively small microgap size.

Rismanchian et al. [66] evaluated microgap size and microbial leakage in the connection area of four different abutments to ITI implants. They reported a microgap size of 74.43 µm for castable (high-noble) abutments [66]. This is greater than the microgap size reported in the present study, which also used castable abutments with high-noble alloys. Per Rismanchian et al.’s conclusion, using pre-machined titanium abutments can reduce the microgap size between implants and abutments compared with Cast-On and castable abutments. However, no differences in the amount of microleakage in their interfaces were found [66]. Piatteli et al. [67] reported a microgap size of 4.33 µm in pre-machined 3i implant abutments. Kano et al. [68] reported vertical misfits of 5.6 µm, 11.1 µm, and 8 µm with pre-machined titanium abutments, Cast-On abutments, and casted Ni-Cr abutments, respectively. In their evaluation, they used optical microscopy and a method similar to that used in the present study [68].

The implant size and the torque used to secure the abutments have been suggested as factors influencing microgap size [29,64]. Furthermore, the casting procedures (which are similar to the ones performed in the present study) may cause distortions and maladaptation between mating surfaces [7]. This is also supported by the conclusion of Kano et al. [69]. Compared to the conventional casting procedures, milling and direct metal laser sintering are preferable [70]. Bending stress as a result of excessive occlusal force (beyond the abutment screw yield strength) directly influences the IAI, leading to abutment screw deformity, which in turn causes IAI separation [71]. The results from the present study suggest that the anti-rotational components in the E abutments did not influence the microgap size in the internal hex implant configurations.

The impact of microgap size and IAI stability has been emphasized in many studies [11,12,13,14,21,22,43,59]; nonetheless, no method of evaluation has been agreed upon or established [72,73]. In contrast to the present study, SEM was used to evaluate the microgap in most of the studies mentioned. Tsunge et al. [5] used a scanning laser microscope (SLM) to compare and evaluate the size of the microgap at the IAI of several external and internal anti-rotation configurations. Although most of the studies conducted to evaluate the IAI and microgap used SEM at different magnifications, the present study used a stereomicroscope with 60× magnification. Several methods used to evaluate the IAI were reported; these include direct view, cross-sectional evaluations, impressions, and the use of a dental explorer combined with visual evaluations [43,72,74,75]. Although cross-sectional evaluations (mostly using SEM) provide more accurate measurements, they require the specimens to be damaged by sectioning; hence, they cannot be used to compare the evaluations made before and after interventions [68], as occurred in the present study. For such interventions, a direct view is convenient when evaluating changes over time [68]. A way to combine the advantages of both direct and cross-sectional methods for evaluating microgaps in in vitro experiments, such as those in the present study, is to use profile projection with optical microscopy [68]. This is a relatively easy method with a lower cost [67]. Optical microscopy permits the use of such repeatable measures [68]. By illuminating the external surface of the IAI and the associated microgap, this technique can be used as a profilometer [68]. This allows direct views and the use of cross-sectional techniques when evaluating the microgap in the in vitro studies [68] that compare the same specimen before and after an intervention (i.e., CL).

Regarding screw surface morphology changes, debris from surface wear between mating surfaces could increase frictional resistance. The method used in this study to evaluate such changes was based on several modifications of the methods used in the previously published studies [61,62], which provided a similar subjective description of changes in the surfaces of implant screws and implant internal threads. Furthermore, the screw material may also be important [75,76]. In this study, titanium alloy Ti-6Al-4V (90% titanium, 6% aluminum, 4% vanadium), in accordance with ISO 5832-3, was used.

Guzaitis et al. [61] reported more titanium debris particles on screw surfaces with increased wear. Arshad et al. [77] reported that with increased loading cycles, smoother surfaces of the crests were found in SEM micrographs [77]. As the cycles were increased, desquamation of the superficial layer was observed in some slopes. Furthermore, after loading, SEM analysis revealed more damage of the thread surface, and some flakes were detected [77]. This is consistent with the findings in the present study, as represented by the qualitative evaluation report. Finally, during SEM analysis, signs of damage were noted, such as non-homogeneity, striations, porosity, and the presence of debris even before cyclic loading with the pristine screws used in this study. This may be attributed to manufacturing errors or damage due to the handling process and is similar to what other studies have reported [61,77,78,79].

Although the evidence from this study suggests that there was no significant difference between the E and NE abutments in terms of microgap size and changes in screw surface morphology, this needs to be interpreted with care given the limitations of this in vitro study. This study has a few limitations. First of all, the in vitro measurements were specific to the system used, which was the NobelBiocare Replace Select Conical Connection with the GoldAdapt abutment. Differences in Morse taper and material likely resulted in variations in the cold welding; consequently, these data cannot be universally applied. However, the system used in this study is in keeping with the most frequently used design of an internal hex taper with an inner wall of 22° [80]. The conical connection, along with the tissue-level design implants, is considered to have the smallest microgap [81]. Secondly, the sample size is small compared to that of the relevant clinical studies. It was noted during data analysis that there was a degree of variation within the dataset represented by limited outliers in the raw data. This might explain the wide range of the standard deviations and the lack of statistical significance. Furthermore, a limitation of in vitro CL studies is the challenge involved in replicating the complex nature of a chewing cycle [77]. Significant effects on the IAI adaptation may be related to clinical parameters, such as intermittent high-impact load, varying angles, and the locations of the load applied [81,82], implant neck design [83], and the site of evaluation relative to the load direction. CL in this study was set to that which seems to be agreed upon as normal masticatory function of approximately 40 months [84,85]. Areas of future research may be directed towards exploring the full three-dimensional context of the microgap throughout the full mating surface at IAC before and after CL using alternative methods such as a Synchrotron-based radiography [86].

## 5. Conclusions

Within the limitations of this study and the implant–abutment system tested, the investigators conclude:

1. No significant difference in microgap size was detected between the E and NE abutments before and after cyclic loading.
2. There was no significant difference in microgap size between the different prosthetic designs before and after CL.
3. From the SEM qualitative evaluation, there were similar, notable screw morphology changes after CL between the E and NE abutments.

This is part of a research project conducted in Kuwait University that yielded two publications:

1. Alzoubi, Fawaz M., et al. “Evaluation of two implant-supported fixed partial denture abutment designs: influence on screw surface characteristics.” *Journal of Prosthodontics* (2023).
2. Alzoubi, Fawaz M., et al. “Preload evaluation of 2 implant-supported fixed partial denture abutment designs.” *The Journal of Prosthetic Dentistry* 128.5 (2022): 1067-e1.

Part of this work was presented as a poster in the 2021 IADR/AADR/CADR General Session (poster ID: 1623).

## Figures and Tables

**Table 1:** Proportions and average microgap sizes over 10 µm for specimens that underwent axial CL.

	Abutment Type	% of Specimens > 10 µm	Microgap Size (M ± SD * µm)
Before Axial CL	Engaging	50.0% (6/12)	15.7 ± 5.4
	Non-engaging	41.7% (10/24)	15.1 ± 3.0
After Axial CL	Engaging	25.0% (3/12)	14.3 ± 1.2
	Non-engaging	41.7% (10/24)	16.4 ± 6.1

* SD: standard deviation. Note: no specimens that underwent lateral CL had microgaps over 10 µm.

**Table 2:** Comparison of microgap sizes (≤10.0 µm versus >10.0 µm) between engaging and non-engaging abutments using chi-square tests.

	Microgap Size				Total		*χ* ^2^	*p*
	≤10 µm		>10 µm					
Before Axial CL								
Engaging Abutments	6	50.0%	6	50.0%	12	100.0%	0.23	0.635
Non-engaging Abutments	14	58.3%	10	41.7%	24	100.0%		
After Axial CL								
Engaging Abutments	9	75.0%	3	25.0%	12	100.0%	0.96	0.326
Non-engaging Abutments	14	58.3%	10	41.7%	24	100.0%		

**Table 3:** Comparison of microgap changes (≤10.0 µm versus >10.0 µm) from before axial CL to after within engaging and non-engaging abutments using McNemar tests of symmetry.

		Microgap Size after Axial CL				Total		McNemar Test (*p*)
		≤10 µm		>10 µm				
Engaging Abutments								
Microgap Size Before Axial CL	≤10 µm	6	100.0%	0	0.0%	6	100.0%	0.250
	>10 µm	3	50.0%	3	50.0%	6	100.0%	
Non-engaging Abutments								
Microgap Size Before Axial CL	≤10 µm	12	85.7%	2	14.3%	14	100.0%	1.000
	>10 µm	2	20.0%	8	80.0%	10	100.0%	

## Data Availability

The original contributions presented in the study are included in the article, further inquiries can be directed to the corresponding author.
